# Sea Buckthorn Leaf Extract Protects Jejunum and Bone Marrow of ^60^Cobalt-Gamma-Irradiated Mice by Regulating Apoptosis and Tissue Regeneration

**DOI:** 10.1155/2015/765705

**Published:** 2015-09-01

**Authors:** Madhu Bala, Manish Gupta, Manu Saini, M. Z. Abdin, Jagdish Prasad

**Affiliations:** ^1^Department of Radiation Biology, Institute of Nuclear Medicine and Allied Sciences, Brig SK Mazumdar Marg, Delhi 110054, India; ^2^Centre for Transgenic Plant Development, Department of Biotechnology, Jamia Hamdard, New Delhi 110062, India

## Abstract

A single dose (30 mg/kg body weight) of standardized sea buckthorn leaf extract (SBL-1), administered 30 min before whole body ^60^Co-gamma-irradiation (lethal dose, 10 Gy), protected >90% of mice population. The purpose of this study was to investigate the mechanism of action of SBL-1 on jejunum and bone marrow, quantify key bioactive compounds, and analyze chemical composition of SBL-1. Study with 9-week-old inbred male Swiss albino Strain ‘A' mice demonstrated that SBL-1 treatment before ^60^Co-gamma-irradiation (10 Gy) significantly (*p* < 0.05) countered radiation induced decreases in jejunum crypts (1.27-fold), villi number (1.41-fold), villus height (1.25-fold), villus cellularity (2.27-fold), cryptal Paneth cells (1.89-fold), and Bcl2 level (1.54-fold). It countered radiation induced increases in cryptal apoptotic cells (1.64-fold) and Bax levels (1.88-fold). It also countered radiation (2 Gy and 3 Gy) induced bone marrow apoptosis (1.59-fold and 1.85-fold) and micronuclei frequency (1.72-fold and 2.6-fold). SBL-1 rendered radiation protection by promoting cryptal stem cells proliferation, by regulating apoptosis, and by countering radiation induced chromosomal damage. Quercetin, Ellagic acid, Gallic acid, high contents polyphenols, tannins, and thiols detected in SBL-1 may have contributed to radiation protection by neutralization of radiation induced oxidative species, supporting stem cell proliferation and tissue regeneration.

## 1. Introduction

The increasing use of ionizing radiation in diagnosis, therapy, industry, and warfare has increased the threat of unwanted exposures of human race to harmful effects of radiation and, therefore, the search for medical radiation countermeasures is an area of intense research [1, 2]. Till this date, no single chemical compound has been approved for human use as a safe, nontoxic, and efficacious radioprotective agent against the lethal dose of radiation. More recently, herbal drugs are gaining better acceptability due to lower toxicity, but most of them are efficacious at sublethal doses of radiation only [3] and are rather ineffective in countering toxicity at lethal doses. The lethal doses of low Linear Energy Transfer (LET) ionizing radiation produce flux of multiple intracellular reactive species and free radicals which severely disturb the redox homeostasis, produce oxidative stress, and damage multiple tissues simultaneously resulting in multiple organ dysfunction/failure, ultimately leading to death.

Nonrecoverable multiple damage to jejunal cells is an important cause of death after radiation exposure at lethal doses. In gastrointestinal (GI) tract, jejunum has sizable number of stem cells as well as proliferating cells and is, therefore, a highly radiation sensitive tissue. The radiation exposure causes depletion of crypts of Lieberkuhn, resulting in loss of villi cellularity, their denudation and shortening, decreased absorption of nutrients, leakage of fluids into the lumen, and increased systemic bacterial infections. Although some herbs were reported to render protection to GI tract from radiation damage, those were found effective at low or sublethal doses of ionizing radiation only [3–5].

Bone marrow is another highly sensitive tissue to the low LET radiation and hematopoietic syndrome occurs >1 Gy, leading to the death of irradiated animals within 3-4 weeks. Bone marrow cells undergo mitotic death or apoptosis after total body exposure to sublethal doses of low LET ionizing radiation (up to 3 Gy). Assessment of bone marrow DNA damage by scoring of micronuclei (MN assay) is one of the established methods for evaluating chromosomal DNA damage caused by ionizing radiations [6]. Micronuclei are small bodies in the cytoplasm resembling the nuclear material in morphology and staining pattern. They are formed when a broken chromosome or chromosome fragment does not travel to the spindle during mitosis and, therefore, are not included in either of the daughter nuclei [6]. Apoptosis is a physiological cell death which is under genetic control. It is characterized morphologically by increased cytoplasmic granularity, cell shrinkage and nuclear and chromosomal condensation, membrane blebbing, and the formation of distinctive nuclear bodies. Cells such as thymocytes, lymphocytes, lymphoblasts, and stem cells undergo apoptosis shortly after irradiation (peaking usually 3-4 h after irradiation) [7]. The process of apoptosis is controlled by a diverse range of cell signals, which may originate either extracellularly (extrinsic inducers) or intracellularly (intrinsic inducers). These signals may positively (i.e., trigger) or negatively (i.e., repress, inhibit, or dampen) affect apoptosis.

The plant* Hippophae rhamnoides *L. (common name sea buckthorn, family* Eleagnaceae*) is a wonder plant loaded with multitude of antioxidants and nutrients. Sea buckthorn is deciduous and dioecious shrub, with silvery leaves and extensive root system, which are very effective in controlling soil erosion. This plant has been adapted to tolerate high radiation stress as well as extreme temperature (−25°C to +40°C), prevalent at high altitude regions in northwest Himalayas (8000–11000 feet) for millions of years. It was argued that, by virtue of adaptation, such a plant may have accumulated unique secondary metabolites, which could be of help in countering radiation toxicity [8]. A large number of bioactive compounds known from this plant, such as carotenoids, a-tocopherols, c-tocopherol, b-tocotrienol, steroids, flavonoids, high amount of unsaturated fatty acids, vitamins A, C, E, and K, minerals, tannins, and polyphenols, contributed to the high antioxidant, hepatoprotective, cardioprotective, wound healing properties [9–11]. Traditionally, sea buckthorn is recommended for treatment of gastric ailments, circulatory disorders, hepatic injuries, and neoplasia [12]. The leaves of sea buckthorn are extremely popular for their nutraceutical as well as medicinal values. Okanagan sea buckthorn tea of Canada has health promoting ingredients such as calcium, magnesium, potassium beta-carotene, flavonoids, lycopene, polyphenols, vitamin E, and protein. Earlier studies demonstrated that sea buckthorn leaves prevented radiation toxicity to experimental mice. Only one time intraperitoneal (i.p.) treatment with 30 mg/kg body weight (b.w.) of SBL-1 (extract from* H. rhamnoides* leaves), 30 min before irradiation with lethal dose of ^60^Co-gamma-rays (10 Gy), rendered 94% survivors in mice population for 30 days and beyond, while all non-SBL-1 treated irradiated (10 Gy) animals died within 12 days after irradiation [8]. SBL-1 treatment before irradiation countered the radiation induced inflammation in mice liver [13] and taste aversion (akin to early nausea and vomiting) in rats [14]. The SBL-1 interacted* in vitro* with DNA and displayed antioxidant properties in a concentration dependent manner [15]. It is proposed that presence of multiple bioactive constituents in a specific proportion was responsible for strong radioprotective effects of SBL-1. The purpose of this study was to provisionally quantify the key bioactive compounds from groups of polyphenols and flavonoids in SBL-1 by using reverse phase high performance liquid chromatography (RP-HPLC), analyze the chemical composition by colorimetric and gravimetric methods, and investigate the mechanism of radioprotective action of SBL-1 by monitoring the bone marrow damage and early (at 48 h) as well as late (up to Day 30) changes in the jejunal histology and levels of key proteins associated with apoptosis using experimental mice as model system.

## 2. Materials and Methods

### 2.1. Reagents and Chemicals

HPLC grade reference standards 3,4,5,-trihydroxybenzoic acid (gallic acid ethyl ester, purity 98%) was purchased from Acros Organics, Fischer Scientific; and 3,3′,4′,5,6-pentahydroxy flavones (quercetin dihydrate, purity 98%) was purchased from Fluka Biochemika. Urea and thiourea were purchased from Hi-Media Laboratories, India. 2,3,7,8-Tetrahydroxy-chromeno[5,4,3-cde]chromene-5,10-dione (ellagic acid), dithiothreitol (DTT), acrylamide, bis-acrylamide, ammonium persulfate, tetramethylethylenediamine (TEMED), sodium dodecyl sulphate (SDS), tris-HCL, phenylmethanesulfonylfluoride (PMSF), 3-[(3-cholamidopropyl) dimethylammonio]-1-propanesulfonate (CHAPS), tween 20, and 3,3′,5,5′-tetramethylbenzidine (TMB) substrate were purchased from Sigma-Aldrich, USA. Polyvinylidene fluoride (PVDF) membranes were purchased from Millipore (India) Pvt., Ltd., India. Mouse monoclonal anti-Bcl-2 primary antibody, anti-beta-actin primary antibody, rabbit polyclonal anti-Bax primary antibody, HRP conjugated anti-mouse IgG secondary antibody, HRP conjugated anti-rabbit IgG secondary antibody, and nonfat dry milk were purchased from Santa Cruz Biotechnology, CA. Methanol, glacial acetic acid, formaldehyde, hematoxylin and eosin stains, and other analytical chemicals and reagents used for HPLC and phytochemical analyses were purchased from Merck Pvt., Ltd., India.

### 2.2. Preparation of Herbal Extract, SBL-1

The SBL-1 was prepared as per procedure described earlier [8]. Briefly, fresh green leaves of* Hippophae rhamnoides *L. (family* Eleagnaceae*), common name sea buckthorn (identified and confirmed by ethnobotanist; the specimen records are preserved at museum, Defence Institute of High Altitude Research, Leh, India (voucher specimen number SBTL-2006)), were collected in the month of September, from a specific natural habitat from western Himalayas, India. The leaves were cleaned, washed thoroughly with distilled water, dried, and powdered. The extract was prepared by soaking the dried leaves powder in distilled water (1 : 1 w/v). The water extract was lyophilized to yield 0.125 gram lyophilized extract, per gram of dried leaves, and was coded as SBL-1 (drug).

### 2.3. Identification and Quantification of Marker Compounds

The HPLC fingerprint of SBL-1 was developed using Agilent HPLC system with quaternary pump attached with PDA detector and Autosampler. EZ Chrome Elite software was used for data computation. A reverse phase C-18 column and mobile phase 0.1% glacial acetic acid : methanol (90 : 10) were used. The detection wavelength was 272 nm. The identification and quantification of marker compounds were performed on the basis of coinjections and matching the retention time with standards. The calibration curves were prepared with standard stocks of 3,4,5,-trihydroxybenzoic acid (gallic acid ethyl ester, purity 98%), 3,3′,4′,5,6-pentahydroxy flavones (quercetin dihydrate, purity 98%), and 2,3,7,8-tetrahydroxy-chromeno[5,4,3-cde]chromene-5,10-dione (ellagic acid). The stock solutions of the standards were prepared in methanol, filtered through 0.22-micron filters (Millipore), and diluted to obtain a suitable concentration for unambiguous identification.

The total phenolic content was determined by the method described [16]. Total flavonoid content was determined by method of [17]. Total tannin content was determined by protein precipitation method [18]. The total thiols were measured as per method of [19].

### 2.4. Experimental Animals

The 8-9-week-old male, inbred, Swiss albino Strain ‘A' mice, weighing 28 ± 2 g, were used after the approval of Animal Experimentation Ethics Committee of the Institute. The animals were maintained under controlled environment at 26 ± 2°C and 12 h light/dark cycle and offered standard animal food (Golden Feed, Delhi) as well as tap water* ad libitum*. All the animal experiments were conducted according to the guidelines of the Committee for Protection and Care of Small Experimental Animals (CPCSEA), Delhi, India.

### 2.5. Experimental Procedure for Studies on Jejunum

The animals were divided into four groups. Group I was untreated control and had three animals, which were administered sterile water (vehicle) only. The other three groups were “treatment groups”; that is, group II animals were whole body ^60^Co-*γ*-irradiated (10 Gy), group III animals were treated with 30 mg/kg body weight (b.w.) SBL-1 (drug) only, and group IV animals were treated with 30 mg/kg b.w. drug, 30 min prior to irradiation (10 Gy). Groups II, III, and IV had 18 animals per group. For whole body irradiation, each mouse was placed in a separate wire mesh container and was given one-time exposure to 10 Gy radiation dose using ^60^Co-*γ*-ray source (GC-220, Atomic Energy of Canada Ltd., Canada, dose rate of 0.31 rad/sec). The fresh air was continuously circulated in the irradiation chamber with the help of pump to avoid hypoxia. For all drug treatments, SBL-1 was dissolved in sterile water, filtered, and administered intraperitoneally (i.p.). A set of three animals were sacrificed from each treatment group at Days 2, 5, 10, 15, and 30 after treatment. From each animal, the jejunum portion of intestine (2-3 cm) was taken out after leaving approximately 4 cm segment from the pyloric sphincter side of stomach. The lumen of jejunum was flushed with normal ice cold saline to remove particulate debris. One half of the jejunum tissue was preserved at −20°C for biochemical analyses while the other half was placed in 10% neutral buffered formalin for histological studies.

### 2.6. Experimental Procedure for Studies on Bone Marrow

The animals were divided into six groups (3 mice in each group) and the experiment was repeated 3 times. Group I was untreated control, in which the animals were administered sterile water only, group II animals were treated with 30 mg/kg b.w. drug only, group III animals were whole body ^60^Co-*γ*-irradiated (2 Gy), group IV animals were whole body ^60^Co-*γ*-irradiated (3 Gy), group V animals were treated with 30 mg/kg b.w. drug, 30 minutes prior to irradiation (2 Gy), and group VI animals were treated with 30 mg/kg b.w. drug, 30 minutes prior to irradiation (3 Gy).

### 2.7. Protein Extraction and Western Blotting

Jejunum was homogenized in lysis buffer containing urea 7 M, thiourea 2 M, DTT 65 mM, CHAPS 0.32 M, and PMSF 2 mM, cell debris was removed by centrifugation, supernatant was collected, and the protein concentration was determined by using Bradford assay [20]. Proteins separation was by electrophoresis on 10% SDS-polyacrylamide gel in 1X SDS PAGE running buffer (25 mM tris base, 192 mM glycine and 0.1% SDS) at 70 V for 4 h at 26 ± 2°C. For western blotting, the gels were fixed (acetic acid, methanol, and Milli Q water: 10 : 50 : 40), washed, and placed in mini Trans blot cell (Bio-RAD, USA) together with PVDF membrane (pore size 0.45 *µ*m). The Trans blot cell was filled with the transfer buffer (25 mM tris base, 192 mM glycine, and 10% methanol) and current was applied at 25 V overnight at 4°C. The membranes were dried and dipped in blocking buffer (5% nonfat skim milk powder) at 4°C for 18 h, treated with primary antibody, and then incubated with HRP conjugated secondary antibodies. TMB solution was used to develop protein bands on the blot. Images were quantified by using image analysis software NIH image J 1.46 software (a free to use application for image analysis, available at  http://rsb.info.nih.gov/ij/)

### 2.8. Tissue Fixation, Staining, and Morphometry

The formalin fixed tissue was dehydrated sequentially with different concentration of alcohol and embedded in paraffin wax. Nonserial transverse sections (approximately 5 *μ*m) of jejunum were cut and stained with hematoxylin and eosin for analysis. Each section was separated from the previous one by a minimum of 50 *μ*m of tissue. Microscopic examinations were done by using light microscope (Axio Scope Observer D1, from Carl Zeiss, Germany).

### 2.9. Surviving Crypt Number, Villi Number, Villi Height, and Villus and Crypt Cellularity

Only complete sections, which included the opening of crypt and full length of villi from base to the tip, were considered for analyses. Villus height was determined by measuring the distance from the tip of the villus up to the crypt in pixels. A surviving crypt was defined as containing 10 or more adjacent, healthy-looking, non-Paneth cells, some Paneth cells, and a lumen [21]. The number of crypts was counted in each circumference. Only those crypts which were seen directly against the inner muscle layer were counted. Cryptal Paneth cells were identified on the basis of their position within the hemispheric bases of the crypts, their truncated shape, basal nuclei, and deep red staining secretion granules and were counted only if they were contained within longitudinally sectioned crypts resting on the muscle layer. All counts and measurements from each tissue specimen were obtained “blind” from a minimum of 4 coded sections. Apoptotic cells were scored within the crypts of jejunum. Apoptosis was assessed on the basis of morphological characteristics such as cell shrinkage, chromatin condensation, and nuclear fragmentation.

### 2.10. Micronuclei Assay

The animals were euthanized by cervical dislocation 30 h after irradiation. The bone marrows from both femurs were flushed in the form of a fine suspension and the assay was done according to Schmid method [22].

### 2.11. Statistical Analysis

The data was presented as mean ± standard deviation (SD). Multivariate analysis of variance (ANOVA) was applied for analyzing statistically important changes in more than one parameter in histological study. Student's *t*-test was applied for evaluating significance between any two treatments, and value at *p* < 0.05 was considered significantly different. Statistical analysis was performed using SPSS software (SPSS Inc., Chicago, IL, USA).

## 3. Results

### 3.1. Total Polyphenols, Flavonoids, Tannins, and Thiols Contents in SBL-1

The SBL-1 (per gram of dried leaves) was found to contain 22 ± 0.2% polyphenolics equivalent to gallic acid, 0.93 + 0.01% flavonoids equivalent to quercetin, 20 ± 1.8% tannins equivalent to tannic acid, and 0.827 M total thiols.

### 3.2. Identification and Quantification of Marker Compounds by RP-HPLC in SBL-1

**Figure 1:**
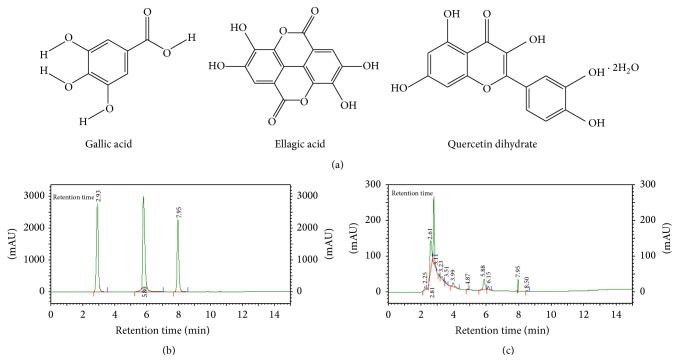
(a) Chemical structure of gallic acid, ellagic acid, and quercetin dihydrate. (b) The HPLC profile of the standards and (c) plant material (SBL-1) using reverse phase C-18 column and mobile phase 0.1% glacial acetic acid : methanol (90 : 10). The detection wavelength was 272 nm.

The structures of marker compounds 3,4,5,-trihydroxybenzoic acid (gallic acid ethyl ester), 3,3′,4′,5,6-pentahydroxy flavones (quercetin), and ellagic acid are shown in Figure 1(a). The RP-HPLC retention time for standard compound gallic acid was 2.93 min, for ellagic acid was 5.80 min, and for quercetin was 7.95 min (Figure 1(b)). The plant extract showed prominent peaks at 2.93 min, 5.80 min, and 7.95 min (Figure 1(c)). The quantitative analysis showed that SBL-1 contained gallic acid: 0.98% w/w, ellagic acid: 0.20% w/w, and quercetin 0.07% w/w.

### 3.3. Effects of Radiation on Tissue Histology, Apoptosis, and Levels of Bcl2 and Bax Proteins

**Figure 2:**
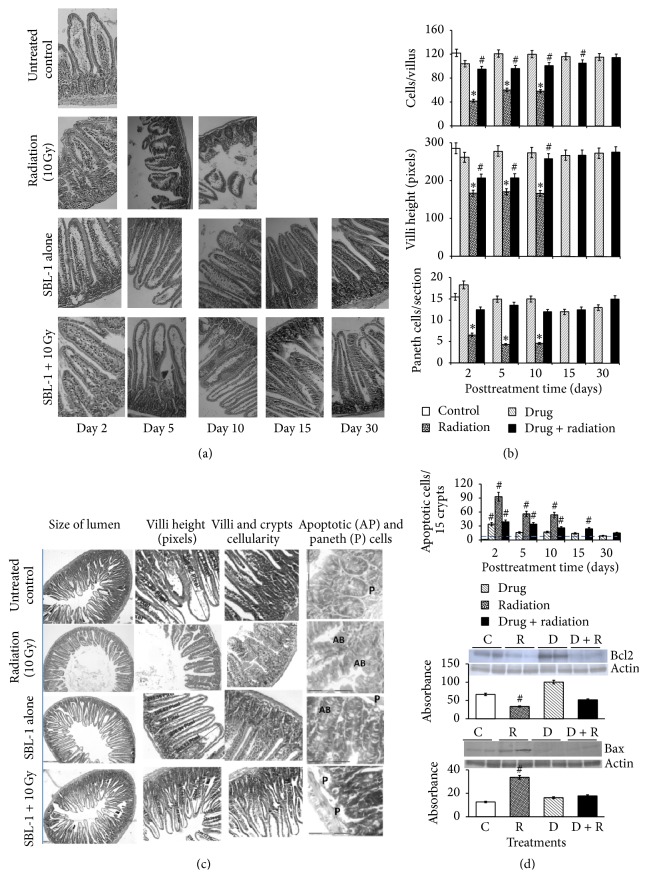
(a) Effects of one-time administration of SBL-1 (30 mg/kg b.w.) 30 min before whole body irradiation (10 Gy), on gross histology of mouse jejunum on Day 2, Day 5, Day 10, Day 15, and Day 30. All the irradiated (10 Gy) animals died by Day 12; in SBL-1 + 10 Gy group 94% of the animals survived, while no death was observed in mice treated with SBl-1 alone [8]. (b) The quantitative changes in the villi height, villus cellularity, and cryptal Paneth cell count from Day 2 till Day 30. (c) The typical radioprotective effects of SBL-1 on Day 5 in terms of jejunal lumen size, villi height, villus and crypt cellularity, and cryptal apoptotic and Paneth cells. (d) Quantitative changes in cryptal apoptotic cells and levels of Bcl2 and Bax proteins on Day 5.

The villi of jejunum from healthy untreated controls were tall and cylindrical. In irradiated animals there were decreases in the villi height, number, and cellularity as well as in crypts number and crypt cellularity with time. At 48 h after irradiation, per section, the crypts number was decreased to 73% and villi number to 63% (significant at *p* < 0.05, in comparison to the untreated control (Table 1)). Also there were other gross histological changes such as villi fusion and nonrecoverable decreases in villi height, villus cellularity, and number of crypts (Figures 2(a) and 2(b)). At Day 5, more sterile crypts and further decreases were observed in villi and crypts cellularity. Significant decreases were also observed in cryptal Paneth cells and Bcl2 protein level, while significant increases were observed in lumen enlargement, cryptal apoptotic cells, and levels of Bax proteins (Figures 2(c) and 2(d)). At Day 10, the jejunal lumen was highly enlarged, villi were nearly absent or severely stunted, and most of the crypts were sterile (Figure 2(a)).

### 3.4. Effects of SBL-1 Treatment on Jejunal Tissue Histology, Apoptosis, and Levels of Bcl2 and Bax

Treatment with SBL-1 30 min before irradiation (10 Gy) countered significantly (*p* < 0.05) the early (within 48 h) radiation induced histological changes. The radiation induced decrease in crypts number was countered by 1.27-fold, villi number by 1.41-fold (Table 1), decreases in villus height by 1.25-fold, villus cellularity by 2.27-fold, and cryptal Paneth cell number by 1.89-fold (Figures 2(a) and 2(b)). At Day 5, the increase in cryptal apoptotic cells was countered by 1.64-fold and levels of Bcl2 and Bax proteins were normalized (Figure 2(d)). The villus and crypts cellularity, villi height, and Paneth cells numbers were further improved (Figure 2(c)). From Day 15 onwards, till the end of the study, there were no significant differences between the SBL-1 treated irradiated animals and untreated controls, in terms of villi and crypts number and villus height. Animals treated with SBL-1 alone did not show any difference from the untreated animals in the jejunum histology as well as in the levels of Bcl2 and Bax.

### 3.5. Percentage Micronuclei Frequency in Bone Marrow

**Figure 3:**
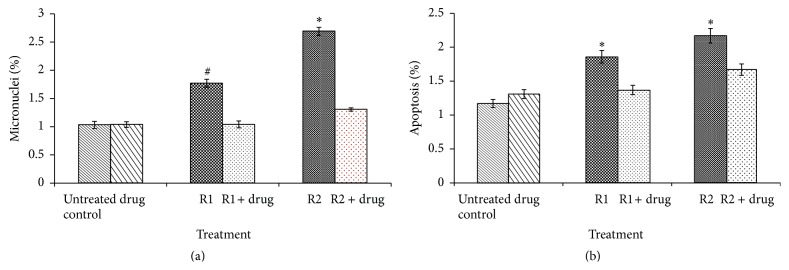
(a) Frequency of micronuclei and (b) apoptosis in bone marrow of mice exposed to 2 Gy (R1) and 3 Gy (R2) ^60^Co-gamma rays with or without SBL-1 (Drug) administration (30 mg/kg/b.wt).

In comparison to untreated control animals, in the irradiated animals, significant increase in micronuclei frequency was observed at 3 Gy (*p* < 0.01, 2.6-fold) and 2 Gy (*p* < 0.01, 1.72-fold) respectively. In drug alone treated animals and drug treated irradiated animals, the micronuclei frequency was comparable to untreated control animals (Figure 3(a)).

### 3.6. Percentage Apoptosis in Bone Marrow

In comparison to untreated control animals, in the irradiated animals, significant increase in apoptosis was observed at 3 Gy (*p* < 0.01, 1.85 folds) and 2 Gy (*p* < 0.01, 1.59 folds) respectively. In drug alone treated animals and drug treated irradiated animals the micronuclei frequency was comparable to untreated control animals (Figure 3(b)).

## 4. Discussion

Radiation causes toxicity and multiple damages to vital biomolecules such as nucleic acids, proteins, and lipids, either by direct deposition of energy or indirectly by generating free radicals and ROS. The resultant effects are radiation dose dependent and may range from cellular losses to the failure of organs. To counter the toxicity caused by lethal doses of radiation, there is a need to identify a pharmacological agent or a group of agents, which have the potential to counter the oxidative stress in multiple manners such as by quenching the high flux of free radicals, breaking the chain reaction of ROS and other reactive species, and preventing lipid peroxidation. Besides these, the agents should essentially have the tissue regenerative properties (such as the potential to recoup cellular loss, regulate apoptosis and prevent inflammation, carry out DNA replication, etc.). The sea buckthorn leaves have nutraceutical, antioxidant, medicinal, and adaptogenic properties [23] and form common constituent of health promoting teas and other beverages. The specific, standardized extract SBL-1, from sea buckthorn leaves, was successful in protecting the mice population from radiation toxicity and injuries in many ways such as increasing the survival of animal population from zero to >90%, restoration of body weight loss, increasing the* in vivo* reducing power of blood plasma [8], and prevention of inflammation by regulating the levels of high mobility group box-1 (HMGB-1) protein, interleukin (IL-10), tumor necrosis factor (TNF-*α*), and immunoglobulin (IgG) [13]. The SBL-1 promoted* in vitro* cell proliferation, had antimutagenic, antirecombinogenic, and antioxidant effects, and demonstrated quenching of radiation induced superoxide radicals, reduction of hydroxyl radicals, promoted metal chelation, and decreased the lipid peroxidation [24].

The polyphenols and flavonoids are important plant metabolites which impart antioxidant properties. The high concentration of polyphenolics (22 ± 0.2%), flavonoids (0.93 + 0.01), tannins (20 ± 1.8%), and thiols (0.827 M) was detected in SBl-1 and explained the strong antioxidant potential of SBL-1. The antioxidant properties of plant polyphenols and flavonoids as well as their beneficial effects have been recently reviewed [25]. Polyphenols are ubiquitous to the plant extracts and may be present as glycosides and esters. They act as free radical scavengers specifically for peroxyl radicals, superoxide anions, and hydroxyl radicals. Flavonoids are a group of compounds (flavonols, anthocyanins, isoflavonoids, flavones, etc.) which have antioxidant properties due to the presence of phenolic hydroxyl groups attached to the ring structure. They act as reducing agents, hydrogen donors, and superoxide radical scavengers. One of the most studied and promising compounds of polyphenols (specifically hydroxybenzoic group) is the gallic acid, which also is the precursor of many tannins [26]. Ellagic acid is a natural phenol antioxidant and is produced in plants by hydrolysis of tannins. Quercetin-3-O-glucoside and Quercetin-3-O-galactoside are common, well-investigated, and important flavonoids present in sea buckthorn leaves [27]. It was, therefore, considered important to quantify the gallic acid, ellagic acid, and quercetin in SBL-1 by HPLC technique. The mobile phase 0.1% glacial acetic acid : methanol (90 : 10) was optimized after many trials to obtain separation of standards as well as of plant constituents (Figures 1(a)–1(c)). The concentrations of gallic acid (0.98% w/w), ellagic acid (0.20% w/w), and quercetin (0.07% w/w) were detected in SBL-1 and explained its multiple effects against radiation toxicity and damage.

In healthy, unirradiated animals, the epithelium of jejunal villi is renewed at regular intervals by newly proliferated cells, which transit from the zone of proliferation to the zone of extrusion at the tip of the villi. The crypts of Lieberkuhn, which are reservoirs of stem cells, are located at the base of villi. The putative multipotent, intestinal stem cells proliferate to increase the production of the clonogenic self-renewing progenitor cells. The daughter cells migrate either toward the villus and differentiate into enterocytes, goblet cells, and enteroendocrine cells or inwards to the crypt base giving rise to Paneth cells. The Paneth cells are important for innate intestinal defense because they act as regulators of microbial density in the small intestine and protect stem cells from infections [28]. Following exposure to ionizing radiation, the cells located at the base of the crypts undergo rapid apoptosis or stop dividing temporarily or permanently depending upon the absorbed radiation dose. If all crypt cells die, the crypt is “sterilized” and ultimately disappears. The loss of regenerating population of clonogenic cells in irradiated animals impairs the normal regeneration of epithelial layer of intestinal villi, leading to varying degrees of blunting, fusion, attenuation, and denudation of the villi. This causes malabsorption, electrolyte imbalance, diarrhea, inflammation, infections, weight loss, and ultimately mortality [29]. At biochemical level, complex interplay of apoptotic proteins such as Bax and p53 as well as antiapoptotic factors such as Bcl2 regulates tissue damage and toxicity [30]. In this study, per section significant decreases in crypts number to 73% and villi number to 63% (Table 1), together with villi fusion and decreases in villi height, villi cellularity, and number of crypts (Figures 2(a) and 2(b)) within 48 h after irradiation, indicated the massive cellular damage to the jejunum by lethal dose of ^60^Co-gamma-irradiation (10 Gy). The appearance of increased number of sterile crypts and further decreases in villus and crypts cellularity at Day 5–Day 10 indicated that most of the stem cells were lost and regeneration of epithelium was not taking place in the irradiated animals. Significant decreases observed in cryptal Paneth cells and Bcl2 protein level and significant increases in cryptal apoptotic cells and levels of Bax proteins suggested that massive apoptosis was progressing with the passage of time (Figures 2(c) and 2(d)). These biochemical as well as histological changes were the typical features of radiation induced GI syndrome and were corroborated with presentations such as diarrheas, weight loss in irradiated animals from Day 1 onwards, and death of all animals by Day 12, reported earlier [8].

The early protection from radiation toxicity by sea buckthorn leaves (SBL-1) was evident because there was significant countering of radiation induced decreases in crypts number, villi number (Table 1), villus height, villus cellularity, and cryptal Paneth cell number (Figures 2(a) and 2(b)) within 48 h. The continuation of protective effects of SBL-1 for subsequent time was observed because, at Day 5, the radiation induced increase in cryptal apoptotic cells was countered, levels of Bcl2 and Bax proteins were normalized (Figure 2(d)), and villi and crypts cellularity, villi height, and Paneth cells numbers were improved (Figure 2(c)). The crypts of Lieberkuhn of GI tract are highly radiosensitive and prone to radiation damage. The early protection (at 48 h) to crypt cellularity against radiation damage could be attributed to effective countering of radiation induced multiple free radicals and reactive species due to the presence of multiple antioxidants (polyphenols, tannins, flavonoids, and thiols), and more specifically gallic acid, ellagic acid, and quercetin in SBL-1. The flavonoids alone were reported to protect from radiation induced oxidative stress but were effective at low or sublethal doses of radiation only [5, 31]. Quercetin alone was also reported to protect the human lymphocytes from genetic damage and oxidative stress caused by low doses of radiation only [32].

The irradiated cells, which undergo DNA damage beyond repair, ultimately undergo apoptosis and show decrease in level of Bcl2 protein and increase in level of Bax protein [33]. Postirradiation increase in apoptosis as well as micronuclei frequency in bone marrow is reported. In jejunum, the radiation induced apoptosis was shown to be accompanied with increased accumulation of Bax and p53 and simultaneous decrease in Bcl2 [30]. Decrease in the frequency of micronuclei as well as apoptotic cells at sublethal doses of radiation (2 Gy and 3 Gy) suggested that the SBL-1 was efficient in countering the radiation damage to hematopoietic system at sublethal doses also. In this study, SBL-1 treated irradiated mice showed the countering of radiation induced apoptosis, with concomitant normalization of Bcl2 and Bax proteins levels. This indicated that SBL-1 acted by countering radiation induced apoptosis as well.

There was no significant difference between the SBL-1 treated irradiated animals and untreated controls, in terms of villi and crypts number and villus height by 15 days and thereafter, suggesting that SBL-1 also acted by promoting the proliferation of surviving cells. The presence of quercetin in SBL-1 may have contributed to this property. The quercetin alone was reported to promote the proliferation of splenocytes in the irradiated mice, although the concentrations required were much higher and the doses were in multiples for 10–30 days [34]. In SBL-1 treated irradiated animals, up to Day 15, significantly greater apoptosis was observed in comparison to untreated controls, though the values were significantly lower than the irradiated controls (Figure 2(d)). This suggested that SBL-1 treatment prior to irradiation permitted controlled and slow apoptosis of cells till Day 15 (Figure 2(d)) and simultaneously promoted proliferation of healthy intestinal stem cells and transition of amplified cells, which ultimately resulted in crypt and villi regeneration (Figures 2(a) and 2(b)). The regulated apoptosis of radiation damaged cells by SBl-1 was expected to be advantageous because increased apoptosis during early time points reduced the burden of damaged nonrepairable cells and hence checked the resultant inflammation and other toxic effects. The presence of flavonoids in SBL-1 could be responsible for altering the regulatory mechanisms because flavonoids have been shown to act by altering the signaling pathways, besides acting as antioxidants [35]. Our* in silico* docking studies had revealed that gallic acid, quercetin, and genistein could block the TWEAK binding site on Fn14 receptors and, therefore, contributed to protection against carcinogenic effects of radiation [36].

The continuous antimicrobial protection of stem cells is of paramount importance and the Paneth cells importantly contribute to the innate intestinal defense [28]. The property of SBL-1 to counter radiation induced decrease in Paneth cell number suggested that SBL-1 was helpful in maintaining the innate immune response of jejunum in the irradiated mice. Treatment with SBL-1 alone did not alter the jejunal histology, or levels of Bcl2 and Bax proteins, significantly, in comparison to the untreated control mice. This demonstrated the nontoxic nature of SBL-1 at the doses used in this study. Other studies have also shown the nontoxic nature of* Hippophae* leaf extract [37].

This study clearly demonstrated that sea buckthorn leaves extract (SBL-1) was effective in preventing radiation toxicity to jejunum. The SBL-1 acted by more than one mechanism such as by reducing oxidative stress, by regulating apoptosis, and by promoting the proliferation of the cryptal stem cells. The radioprotective effects of SBL-1 on jejunum were higher than those observed with most investigated single molecular drugs WR 2721 and MPG alone or in combination [38]. The SBL-1 treatment demonstrated the dose reduction factor of 1.41 on the basis of whole body survival and 1.97 on the basis of endogenous spleen CFUs counts; the effective SBL-1 concentration was 4 times lower than the maximum tolerated dose (MTD) [8]. In this manner, SBL-1 met with two essential criteria of an ideal radioprotector in being nontoxic and effective at lethal doses of whole body irradiation. The radioprotective survival effect of SBL-1 against lethal doses of ionizing radiation was better than the other preparations from sea buckthorn such as SBL-2 from leaves [39] and RH-3 from berries [40], which caused only 82% survival at 3/4 of MTD [41]. The sea buckthorn leaf extract SBL-1 displayed a number of other distinct advantages over the sea buckthorn berry extract RH-3, such as longer shelf life, lower storage and transportation cost, higher stability of active constituents, better ecofriendly collection of plant material, and zero destruction of plant germplasm. To the best of our knowledge, this is the first report presenting the protective effects of sea buckthorn leaves on jejunum in lethally irradiated experimental animals.

## 5. Conclusion

The manifestation of gastrointestinal syndrome, at doses above 6 Gy, is the common cause of death of irradiated animals. The study showed that single prophylactic dose of SBL-1 (30 mg/kg body weight) before lethal irradiation (10 Gy) countered the radiation induced atrophy of mucosal layer, decrease in jejunum villi number and cellularity, crypts number and caused normalization of apoptosis and levels of Bcl2 and Bax expression. These observations suggested a strong radioprotective action of SBL-1 on the jejunum of lethally irradiated experimental mice. The damage suffered by bone marrow above 1 Gy is another important cause of death of irradiated animals. This study showed that single prophylactic dose of SBL-1 at 30 mg/kg body weight before sublethal irradiation (2 Gy and 3 Gy) significantly countered the apoptosis, as well as formation of micronuclei in bone marrow, therefore suggesting the radioprotective action of SBL-1 on hematopoietic system. Sea buckthorn leaves form a part of dietary constituents in many parts of the world because of their nutraceutical properties. This study, together with earlier published reports, which demonstrated the radioprotective action of sea buckthorn on immune system through HMGB1 modification in irradiated (10 Gy) mice, action of SBL-1 to counter taste aversion (akin to early nausea and vomiting) in irradiated (2 Gy) rats by normalizing jejunal serotonin levels, antimutagenic and antirecombinogenic action of SBL-1 in* Saccharomyces cerevisiae*, and significant (>90%) whole body radioprotection at nontoxic doses with dose reduction factor > 1.4, suggested that SBL-1 could be developed as a promising medical radiation countermeasure for human use.

## Figures and Tables

**Table 1:** Effects of SBL-1 (30 mg/kg b.w., −30 min) before irradiation (10 Gy) on villi and crypts number of mouse intestine, at 48 h after irradiation (10 Gy).

Treatment	Villi number/section	Crypts number/section
Untreated control	46 ± 0.91	105 ± 3
SBL-1 alone	45 ± 0.87	102 ± 4
10 Gy	29 ± 0.4^*∗*^	77 ± 2^*∗*^
SBL-1 + 10 Gy	41 ± 0.5	98 ± 3

^*∗*^Significantly reduced at *p* < 0.05 in comparison to untreated control.
